# Comparison of scar outcomes of alar flare region using absorbable and non-absorbable sutures: a single-blind study

**DOI:** 10.1016/j.bjorl.2022.06.001

**Published:** 2022-06-15

**Authors:** Mehmet Emrah Ceylan, Hasan Hüseyin Balıkçı

**Affiliations:** a Isparta Davraz Yasam Hospital, Department of Otolaryngology, Isparta, Turkey; b Private Otolaryngology Clinic, Antalya, Turkey

**Keywords:** Polyglactin 910, Polypropylenes, Rhinoplasty, Cicatrix, Surveys and questionnaires

## Abstract

**Objective:**

Formation of scar on the face after septorhinoplasty may disturb the patient due to cosmetic concerns. One of the main factors affecting scar outcomes is probably the suture material used. The aim of this study was to examine the effect of different suture materials on scar outcomes of alar base in patients undergoing septorhinoplasty.

**Methods:**

Thirty-one patients who underwent alar base intervention during primary septorhinoplasty were divided into two groups according to the suture material used as the Polypropylene group (n = 16), (Polypropylene, Prolene 6/0; Ethicon Inc., Somerville, NJ, USA) and the Polyglactin group (n = 15), (Irradiated polyglactin 911, Vicryl Rapid™ 6/0; Ethicon Inc., Somerville, NJ, USA). The scar outcomes of alar base were compared between the Polypropylene and Polyglactin groups. The modified Stony Brook Scar Evaluation Scale was used to measure wound healing results at one and 12-months postoperatively for objective evaluation. Patient satisfaction questionnaire was used for subjective evaluation.

**Results:**

There were no statistically significant differences in the Stony Brook Scar Evaluation Scale and patient questionnaire scores between Polypropylene and Polyglactin groups. Irradiated Vicryl Rapid had poor cosmetic outcomes in the alar base when compared to polypropylene, indicating no statistically significant difference.

**Conclusion:**

Both sutures can be used for closure of alar base considering their advantages and disadvantages, in patients undergoing septorhinoplasty.

**Level of evidence:**

Treatment Benefits; Level 2 (Randomized Trial).

## Highlights

• During septorhinoplasty, alar base interventions are performed frequently.
• One of the major factors affecting the scar outcome is the suture material used.
• Absorbable sutures were reported to provide favorable cosmetic outcomes on skin wounds.
• There is no study investigating scar outcomes of alar base using different sutures.
• Vicryl rapid has worse scar outcomes on alar base compared to Polyproplyene.

## Introduction

During Septorhinoplasty (SRP), alar base interventions are performed frequently, when the interalar distance exceeds the intercanthal distance or the lateral aspect of the ala extends considerably beyond the alar-facial groove.^1^ Formation of scar on the face may disturb the patient due to cosmetic concerns. Factors such as the incision and closure technique or postoperative care play a key role in the formation of visible scars after SRP.^2^, ^3^, ^4^ One of the major factors affecting the scar outcome is probably the suture material used. Although the use of prolene suture is classically preferred in the alar flare region,^1^, ^2^, ^3^, ^5^ absorbable suture is preferred by some surgeons recently, as it eliminates the need for much more suture removal and saves office time.

In a meta-analysis of 10 randomized-controlled studies on sutures, absorbable and non-absorbable sutures were found to be similar in terms of operative morbidities, and there was insufficient data on cosmetic outcomes.^6^ Although absorbable sutures were reported to provide favorable cosmetic outcomes on skin wounds,^7^, ^8^ there are still opposing views in the literature.^9^

To the best of authors’ knowledge, there is no study investigating scar outcomes of alar flare region using different sutures in the literature. In the present study, the objective was to investigate the scar outcomes using absorbable and non-absorbable sutures in the alar flare region in early- and long-term healing period after SRP.

## Methods

### Study design and study population

This single-center, retrospective study was conducted at the ear, nose, and throat clinic. The study protocol was approved by the Süleyman Demirel University Ethics Committee (03/08/2021 - 16/257). A written informed consent was obtained from each patient for all diagnostic and therapeutic procedures. The study was conducted in accordance with the principles of the 1964 Declaration of Helsinki and its later amendments.

The patients who underwent wedge resection for correcting alar flare during primary SRP between January 2018 and July 2021 were reviewed and 54 patients invited to the clinic by phone. Revision cases and cases with nostril sill excision were excluded from the study. The patients who attended the invitation, agreed to participate in the study and had postoperative photographic documentations at first and twelfth month were included in the study. Finally, 31 patients were divided into two groups according to the suture material used on the alar flare region as the PP group (Polypropylene), (n = 16), and the PG group (Irradiated polyglactin 911), (n = 15). All surgeries and closures performed by senior author and irradiated polyglactin 911 (Vicryl Rapid™ 6/0; Ethicon Inc., Somerville, NJ, USA) was used since June 2019 for closure of the alar flare region incisions. Before this date, polypropylene (Prolene 6/0; Ethicon Inc., Somerville, NJ, USA) was preferred. All patients were photographed in a routine six-way fashion, under same conditions, using a professional camera (Epl-1; Olympus Imaging Corp., Shinjuku, Tokyo, Japan), lens (Micro 60 mm/F 2,8; Sigma Corp., Kawasaki, Japan) and double para flash (TT560 Speedlite; Neewer, Shenzhen, China) preoperatively and at postoperative follow-ups. Patient satisfaction survey questionnaire was applied to all patients during their scheduled follow-up. Retrospective examination was made on the photographs of the patients taken at postoperative one and 12-months.

### Assessment tools

All patients were asked during their scheduled follow-up visit to grade their postoperative alar flare region scars using a satisfaction survey questionnaire, as previously described for the evaluation of columellar scar.^10^ The survey consists of the following questions: “What do you think about your scar at the alar flare region area after the rhinoplasty operation?” (1 ‒ I am not satisfied at all, 2 ‒ I am not satisfied, 3 ‒ I am neutral, 4 ‒ I am satisfied, 5 ‒ I am very satisfied) and “Did you need to hide this scar?” (1 ‒ Always, 2 ‒ Frequently, 3 ‒ Sometimes, 4 ‒ Very rare, 5 ‒ Never) (0 worst/10 best).

In this study, it was planned to use the Stony Brook Scar Evaluation Scale (SBSES), which has been shown to be a valid and reliable tool for scar evaluation, for obtaining an objective examination.^11^ This scale consists of five items: width, height, color, hatch/suture marks, and overall appearance.^12^ Verim et al.^13^ modified this scale for columellar scar evaluation by adding notching instead of hatching to the SBSES and reported that the aim of this change was to examine the negative effect of wound tension on scar. However, Erol et al.,^10^ in their prospective study about columellar scar, used a different assessment scale derived from the Manchester and Vancouver scar scales. Using this scale, short-term inflammation signs, such as pigmentation and hyperemia, were evaluated by grading discoloration as hyperpigmentation, slight pigmentation, and normal skin color. In the current study, the modified SBSES in which hatching was added instead of scar width was used, as a ruler was not used while photographing. In addition, color was graded as darker (hyperpigmentation), slightly darker (slight pigmentation), and normal or lighter from surrounding skin to compare signs of inflammation between the groups at one and 12-months postoperatively (Table 1).

**Table 1:** Modified stony brook scar evaluation scale.

Scar category	Points
Height	
Depressed or elevated from surrounding skin	0
Flat	1
Color	
Darker than surrounding skin (Hyperpigmentation)	0
Slightly Darker than surrounding skin (Slight pigmentation)	1
Same color or lighter	2
Notching	
Present	0
Absent	1
Hatching	
Present	0
Absent	1
Overall appearance	
Poor	0
Good	1

Total scar score: sum of individual scores; range, 0 (worst) to 6 (best).

Patients' skin types were analyzed according to the Fitzpatrick skin type classification to evaluate the amount of melanin pigment in the skin, which is one of the key predictors of pigmentation in the scar.^13^, ^14^

Another otolaryngologist (A.A.), who specializes in rhinoplasty and blind to the type of suture used in each case, used the modified SBSES to examine the postoperative one- and twelve-month photographs of the alar flare region in all patients. The scars were assigned 0 or 1 point each for the presence or absence of the following: elevation or depression, notching, hatching and overall appearance. Color was graded between 0 (worst) and 2 (best) points. A total cosmetic score was, then, calculated by adding the individual scores for each of the five categories ranging from 0 (worst) to 6 (best). Total scar scores were categorized into three groups: poor (0‒1 point), moderate (2‒3 points), and good (4‒6 points). Patient questionnaires and postoperative one- and twelve-month modified SBSES scores were analyzed.

### Surgical technique

**Figure 1:**
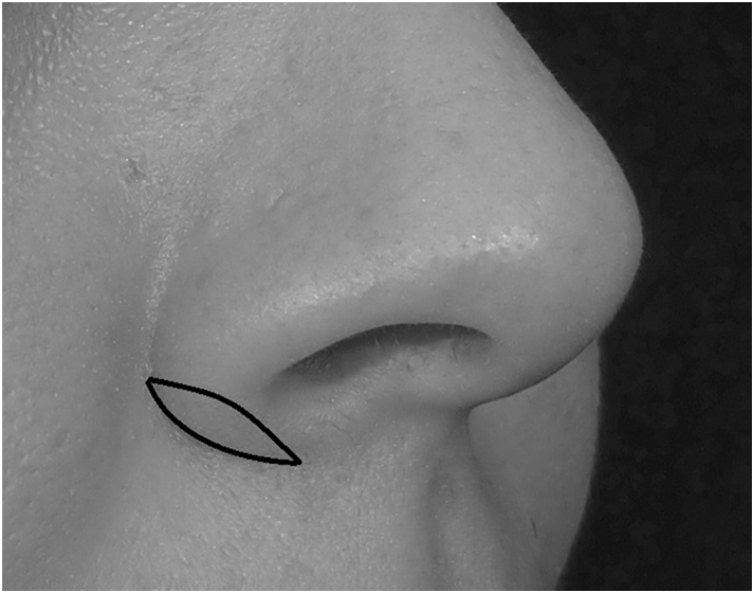
Modified Weir incision on the alar-facial groove.

After local anesthetic injection (1% lidocaine with 1:100,000 epinephrine) for hemostasis, a modified Weir incision was used in which the lateral incision was placed at the level of the alar-facial groove and wedge resection was done in all patients (Fig. 1). This surgical technique was used in the same way in all patients to hide the incision in the alar-facial groove. Incisions were made with saw-like movements, and the deep tissues up to the underlying muscles were excised. After bipolar cauterization, simple interrupted PG or PP sutures were used to close the wound edges. All patients were instructed to apply hydrogen peroxide followed by topical mupirocin ointment to the incision lines and sutures twice daily for seven days to prevent crusting. The PP sutures were removed on postoperative Day 7. The PG sutures did not trim soon, and none of the patient’s received treatment with external scar-revising prescriptions after surgery. There was no patient with a history of hypertrophic scar and/or keloid on the skin.

### Statistical analysis

Statistical analysis was performed using the SPSS version 25 software (IBM Corp., Armonk, NY, USA). Continuous variables were presented in mean ± Standard Deviation (SD), while categorical variables were presented in n and frequency. The Kolmogorov-Smirnov and Shapiro-Wilk tests were used for normality tests. The Mann-Whitney *U* test was used for group differences for continuous variables and the Fisher exact test was used for categorical variables. A *p*-value of <0.05 was considered statistically significant.

## Results

Of a total of 31 patients included in the study, 1 (7%) was male and 14 (93%) were females in the PG group and 2 (12.5%) were males and 14 (87.5%) were females in the PP group. There was no statistically significant difference between the two groups in terms of sex distribution (*p* =  0.52). The mean age was 26.5 ± 8.5 (range, 20–54) and 29.5 ± 9.8 (range, 19–56) years in the PG and PP groups, respectively. There was no statistically significant difference in the mean age of the patients between the PG and PP groups (*p* =  0.45). Wound infection, dehiscence or significant wideness of the scar were not seen in either group. The mean time to patient questionnaire was 16.9 ± 5.2 and 20.1 ± 7.6 months in the PG and PP groups, respectively (*p* =  0.18).

The mean total score corresponding to Fitzpatrcik skin type of the groups was 22.6 ± 6.5 and 22.7 ± 6.1 in the PG group and PP group, respectively. There was no statistically significant difference in the total scores of Fitzpatrcik skin type of the patients between the PG and PP groups (*p* = 0.95). The distribution of the patients according to the Fitzpatrick skin type scale and the *p*-values are detailed in Table 2. The mean patient questionnaire total score was 8 ± 2.4 and 9.2 ± 1.4 in the PG group and PP group, respectively (Table 3). There was no statistically significant difference in the patient questionnaire scores between the PG and PP groups (*p* = 0.35).

**Table 2:** The distribution of the patients according to the Fitzpatrick skin type scale and the *p*-values.

Fitzpatrick skin type	PP group, n (%)	PG Group, n (%)	p
Type 2	3 (19)	4 (27)	0.68
Type 3	5 (31)	3 (20)	0.68
Type 4	8 (50)	8 (53)	0.85

PP, Polypropylene; PG, Polyglactin.

**Table 3:** Patient questionnaire of groups according to the suture material.

	PP Group, n = 16 (mean ± SD)	PG Group, n = 15 (mean ± SD)	p
Questionnaire time	20.1 ± 7.6 months	16.9 ± 5.2 months	0.18
Satisfaction	4.6 ± 0.7	3.7 ± 1.3	0.06
Need to hide	4.6 ± 0.7	4.2 ± 1.2	0.31
Total score	9.2 ± 1.4	8 ± 2.4	0.07

PP, Polypropylene; PG, Polyglactin.

The mean modified SBSES score was 4.7 ± 1 and 4.8 ± 1.2 at one month in the PG and PP group, respectively. At twelve months, the mean scores were 5 ± 1.3 and 5.3 ± 0.9 in the PG and PP group, respectively. There was no statistically significant difference in the modified SBSES scores at one and twelve months between the PG and PP groups (*p* =  0.66, *p* =  0.84). Detailed SBSES scores of the groups are shown in Table 4. There were two patients (6%) who considered dermabrasion for their scar revision. Both of these patients were in PG group.

**Table 4:** Modified Stony Brook Scar Evaluation Scale outcomes of alar flare region in PP and PG groups.

SBSES	PP Group (n = 16)	PG Group (n = 15)		PP Group (n = 16)	PG Group (n = 15)	
	n (%)	n (%)		n (%)	n (%)	
	Postop. Month 1	Postop. Month 1	p	Postop. Month 12	Postop. Month 12	p
Height						
Depressed/elevated	3 (19)	2 (13)	0.53	2 (12)	1 (7)	0.52
Flat	13 (81)	13 (87)	0.53	14 (87)	14 (93)	0.52
Discoloration						
Hyperpigmentation	2 (12)	0	0.25	1 (6)	0	0.51
Slight pigmentation	10 (62)	12 (80)	0.25	1 (6)	2 (13)	0.47
Same color or lighter	4 (25)	3 (20)	0.53	14 (87)	13 (86)	0.67
Notching						
Present	0	2 (13)	0.22	5 (31)	4 (27)	0.54
Absent	16 (100)	13 (86)	0.22	11 (69)	11 (73)	0.54
Hatching						
Present	0	2 (13)	0.22	0	1 (7)	0.48
Absent	16 (100)	13 (87)	0.22	16 (100)	14 (93)	0.48
Overall appearance						
Poor	2 (12)	1 (7)	0.52	1 (6)	3 (20)	0.27
Good	14 (87)	14 (93)	0.52	15 (94)	12 (80)	0.27
SBSES Group						
Poor	0	0	–	0	0	–
Moderate	2 (12)	2 (13)	0.67	1 (6)	3 (20)	0.27
Good	14 (87)	13 (87)	0.67	15 (94)	12 (80)	0.27
SBSES Score	(mean ± SD)			(mean ± SD)		
Total	4.8 ± 1.2	4.7 ± 1	0.66	5.3 ± 0.9	5 ± 1.3	0.84

Postop., Postoperative; PP, Polypropylene; PG, Polyglactin.

## Discussion

In the present study, scar outcomes of alar flare region using PP and irradiated PG sutures were compared in patients undergoing SRP. The early- and long-term assessment of the modified SBSES and long-term patient questionnaire scores did not significantly differ according to the use of absorbable or non-absorbable suture materials. To the best of authors’ knowledge, this is the first study to examine scar outcomes of alar flare region using different sutures in the literature.

Wound healing is a complex process consisting of inflammation, proliferation, and maturation phases. It has been reported that the time it takes to heal a wound at the age of 20 can be doubled for a 40-year-old individual.^15^ In addition, the increased incidence of comorbidities with age may lead to delayed wound healing and poor scar quality. In the current study, there was no statistically significant difference between the groups in terms of age.

In the PP group, the patient questionnaire scores were higher. The modified SBSES scores of the PP group at one and twelve months were also better than the PG group. The outcomes of early- and long-term modified SBSES scores and long-term patient questionnaire scores were comparable. Although better results were achieved with the use of PP sutures in the alar flare region, there was no statistically significant difference between the groups. More intriguingly, on detailed examination of the patient questionnaire, the PG group had considerably lower ‘satisfaction’ scores compared to the PP group. Despite this low score, the PG group had a closer score in ‘need to hide’ item to the PP group. This may be a reflection of the increased self-confidence of the patients after SRP.

Verim et al.^13^ reported that skin thickness and types were not determinants of columellar scar healing in Turkish population. In our study there was no statistically significant difference in terms of skin types of distribution in the PP and PG groups. However, since this was not the aim of this study, a comparison between skin types and SBSES scores on the alar flare region was not made.

Kriedel et al.^1^ reported that increased density of sebaceous glands in the area of the alar-facial groove undoubtedly played a role in the visibility of Weir incisions. It has been proposed that this sebaceous nature may lead to a predisposition to epithelial cysts and micro-abscess formation during healing period, which may cause to a visible scar. Also, a large number of muscles have been described that can cause wound tension in the alar flare region. The dilatator naris muscle and the alar portion of the nasalis muscle insert directly onto the alar skin,^16^ and the levator labii superioris partially inserts on the vestibular skin of the nasal vestibule.^17^ Daniel et al.^18^ showed that lower nasal base was not an isolated, static structure, but rather a very dynamic form integrated into the nasal superficial musculoaponeurotic system and even the entire facial musculature. In addition, Parell et al.^7^ found in their study about scar outcomes of facial skin wounds, that a wound over a bony structure might be with an increased wound tension. The edges of the alar flare reduction may be subject to severe traction due to the many surrounding muscles and the bony structure underneath. These anatomical and physiological features may cause poor scar outcomes and satisfaction due to the increased wound tension in patients with alar flare reduction.

The PP sutures are non-absorbable, monofilament, unlikely to break prematurely, and elicit a minimal inflammatory response. However, due to the springy nature of these sutures, there may be difficulties in tying with a tendency to unravel. Also, suture removal may cause patient discomfort and waste of office time. The PG sutures are absorbable, easier to tie, and do not need to be removed, thereby reducing patient discomfort, and saving office time. On the other hand, due to their absorbable and braided nature, they may cause inflammation and tend to promote infection.^7^, ^10^ In two animal experiments and a research article which examines full thickness skin grafts, low inflammation and local reactions were observed with the use of PG.^19^, ^20^, ^21^ Parell et al.^7^ found no significant difference in the long-term cosmetic outcomes on facial skin wounds between the PP and PG use. On the other hand, there is one study reporting that color change and hypertrophic scar lasting up to one year were detected with the use of PG in inframammary skin closure.^22^ In current study, the inflammation signs were compared by grading color changes in the early- and long-term period. There was no statistically significant difference in the early- and long-term inflammation signs between the PP and PG groups.

**Figure 2:**
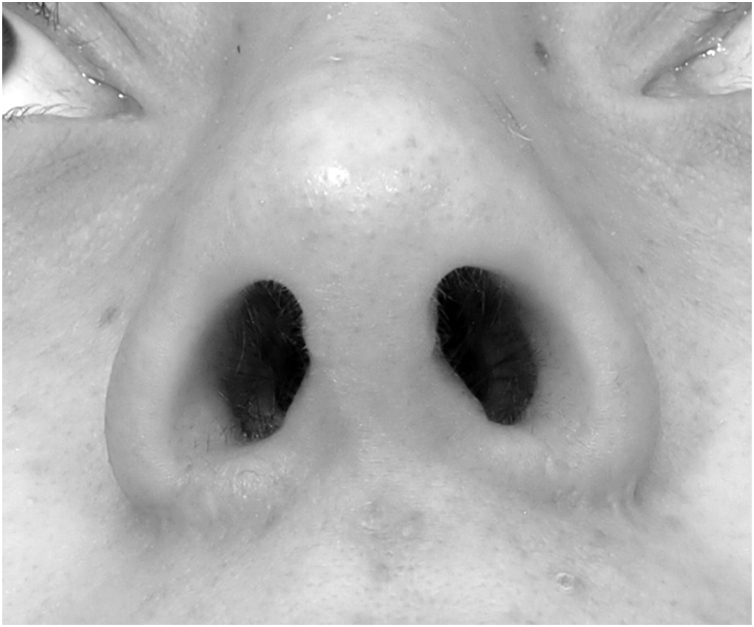
Hatching at right side of the patients’ alar flare region. Notching at left side of the patients’ alar flare region at 12-months of follow-up.

Dermabrasion is the most accessible and most studied technique for scar resurfacing. This procedure improves wound contour and color match to surrounding skin using sterile sandpaper or diamond burs.^23^ In the literature, the rate of dermabrasion after alar flare reduction has been reported as 0%–25%.^1^, ^2^, ^3^, ^24^ In the present study, the patients who considered dermabrasion for their scar was 6% (2/31). Among these, two patients in the PG group, one had hatching (Fig. 2) and the other had depressed scar and notching.

As described by Kriedel et al.,^1^ an important factor to contribute to notching is carrying the modified Weir incision into the deep muscle layer in the alar flare region. During alar region interventions, adhering a conservative plan with basic surgical principles such as precise and right-angle cut on the skin, eversion of the wound edges during closure and meticulous postoperative care may provide good scar outcomes. Also, stitches should not be too tight to prevent hatching. During postoperative care, antibiotic ointment and meticulous cleaning of clustering should be applied. Therefore, meticulous attention should be paid during alar region interventions and in postoperative care.

The main strength of this study is that it examines the effect of selected suture material on scar outcomes in alar flare region at early- and long-term period based on subjective and single-blinded objective methods. Main limitation of this study is its retrospective nature, small sample size. Statistical power analysis could not have performed on the data set due to small sample size. Although the patients evaluated regarding their skin type, no comparison was made between the groups in terms of skin thickness. Last limitation of this study is that genetic, environmental, and systemic factors such as diabetes mellitus, smoking and hypoproteinemia that may adversely affect wound healing apart from age were unable to be evaluated among the groups.

## Conclusions

In conclusion, the study results showed that, although not statistically significant, PG has worse scar outcomes on alar base compared to PP. This study is valuable as it contributes to the literature with examining the effect of different suture materials on the cosmetic outcomes of the alar flare region. It is concluded that both PG and PP sutures can be used for closure considering their advantages and disadvantages, in patients with alar flare reduction. Nevertheless, further large-scale, prospective, randomized studies are needed to draw more reliable conclusions on this subject.

## Funding

This study received no specific grant from any funding agency in the public, commercial or not‐for‐profit sectors.

## Consent

Prior to surgery, all patients were informed, and a written informed consent was obtained for all diagnostic and therapeutic procedures. An additional consent was obtained from the patient for the publication of images in Figure 1, Figure 2.

## Conflicts of interest

The authors declare no conflicts of interest.

## Acknowledgement

Thanks to Prof. Dr. Murat Songu for his valuable advices and technical editing.

## References

[1] Kridel R.W., Castellano R.D. (2005). A simplified approach to alar base reduction: a review of 124 patients over 20 years. Arch Facial Plast Surg.

[2] Hudise J.Y., Aldhabaan S.A., Nassar R.S., Alarfaj A.M. (2020). Evaluation of scar outcome after alar base reduction using different surgical approaches. J Oral Maxillofac Surg..

[3] Warner J.P., Chauhan N., Adamson P.A. (2010). Alar soft-tissue techniques in rhinoplasty: algorithmic approach, quantifiable guidelines, and scar outcomes from a single surgeon experience. Arch Facial Plast Surg..

[4] Aksu I., Alim H., Tellioğlu A.T. (2008). Comparative columellar scar analysis between transverse and inverted-V incision in open rhinoplasty. Aesthetic Plast Surg..

[5] Carniol E.T., Adamson P.A. (2018). Surgical tips for the management of the wide nasal base. Facial Plast Surg..

[6] Sajid M.S., McFall M.R., Whitehouse P.A., Sains P.S. (2014). Systematic review of absorbable vs non-absorbable sutures used for the closure of surgical incisions. World J Gastrointest Surg..

[7] Parell G.J., Becker G.D. (2003). Comparison of absorbable with nonabsorbable sutures in closure of facial skin wounds. Arch Facial Plast Surg..

[8] Rosenzweig L.B., Abdelmalek M., Ho J., Hruza G.J. (2010). Equal cosmetic outcomes with 5‒0 poliglecaprone-25 versus 6‒0 polypropylene for superficial closures. Dermatol Surg..

[9] Niessen F.B., Spauwen P.H., Kon M. (1997). The role of suture material in hypertrophic scar formation: Monocryl vs. Vicryl-rapide. Ann Plast Surg..

[10] Erol O., Buyuklu F., Koycu A., Jafarov S., Gultekin G., Erbek S.S. (2020). Comparison of rapid absorbable sutures with nonabsorbable sutures in closing transcolumellar incision in septorhinoplasty: short-term outcomes. Aesthetic Plast Surg..

[11] Nguyen T.A., Feldstein S.I., Shumaker P.R., Krakowski A.C. (2015). A review of scar assessment scales. Semin Cutan Med Surg..

[12] Singer A.J., Arora B., Dagum A., Valentine S., Hollander J.E. (2007). Development and validation of a novel scar evaluation scale. Plast Reconstr Surg..

[13] Verim A., Duymuş R., Çalim ÖF. (2013). Effect of nose skin on the columellar incision scar in a Turkish population. Otolaryngol Head Neck Surg..

[14] Sachdeva S. (2009). Fitzpatrick skin typing: applications in dermatology. Indian J Dermatol Venereol Leprol..

[15] Beyene R.T., Derryberry S.L., Barbul A. (2020). The effect of comorbidities on wound healing. Surg Clin North Am..

[16] Bruintjes T.D., van Olphen A.F., Hillen B., Huizing E.H. (1998). A functional anatomic study of the relationship of the nasal cartilages and muscles to the nasal valve area. Laryngoscope..

[17] Rohrich R.J., Hoxworth R.E., Thornton J.F., Pessa J.E. (2008). The pyriform ligament. Plast Reconstr Surg..

[18] Daniel R.K., Glasz T., Molnar G., Palhazi P., Saban Y., Journel B. (2013). The lower nasal base: an anatomical study. Aesthet Surg J..

[19] Gazivoda D., Pelemiš D., Vujašković G., Djurdjević S. (2015). Influence of suturing material on wound healing - An experimental study on dogs. Vojnosanit Pregl..

[20] Brackeen A.R., Wells M.J., Freed J.M. (2005). Irradiated polyglactin 910 (Vicryl Rapide) for placement of full-thickness skin grafts. Dermatol Surg..

[21] Gartti-Jardim E.C., de Souza A.P., Carvalho A.C., Pereira C.C., Okamoto R., Magro Filho O. (2013). Comparative study of the healing process when using Vicryl®, Vicryl Rapid®, Vicryl Plus®, and Monocryl® sutures in the rat dermal tissue. Oral Maxillofac Surg..

[22] Niessen F.B., Spauwen P.H., Kon M. (1997). The role of suture material in hypertrophic scar formation: Monocryl vs. Vicryl-rapide. Ann Plast Surg.

[23] Ward R.E., Sklar L.R., Eisen D.B. (2019). Surgical and noninvasive modalities for scar revision. Dermatol Clin.

[24] Foda H.M. (2007). Nasal base narrowing: the combined alar base excision technique. Arch Facial Plast Surg.

